# Supplementary material for: DNA Damage Response Alterations in Ovarian Cancer: From Molecular Mechanisms to Therapeutic Opportunities
Source: Cancers (Basel). 2023 Jan 10;15(2):448. doi: 10.3390/cancers15020448 (PMC9856346; doi:10.3390/cancers15020448)
Supplement: Supplementary file 1 [file cancers-15-00448-s001.zip › Table S1 PARPi.pdf]

Drug	Combined with	Condition	Phase	Clinical trial ID	Results
Olaparib	-	*BRCA*-mutated platinum-sensitive HGSC OC	Phase I	NCT02855697	No results posted
		BRCA-mutated OC	Phase I	NCT03943173	No results posted, recruiting patients
		BRCA-mutated recurrent OC	Phase I	NCT05258747	No results posted, recruiting patients
			Phase I	NCT04041128	No results posted
		OC	Phase I	NCT03378297	No results posted, recruiting patients
		OC	Phase II	NCT02489006	No results posted
		Relapsed OC	Phase II	NCT02822157	Olaparib statistically showed a favorable response rate in heavily pre-treated relapsed OC compromising mainly platinum-resistant and patients with *BRCA* mutations [294]
		Recurrent OC	Phase II	NCT04780945	No results posted
				NCT04377087	No results posted
				NCT03470805	No results posted
		BRCA-mutated recurrent OC	Phase II	NCT00679783	Olaparib demonstrated promising results in recurrent OC patients with *BRCA* mutations [295]
		BRCA-mutated advanced OC	Phase II	NCT01078662	Olaparib demonstrated notable antitumor activity in heavily pre-treated OC patients with advanced tumors that harbored *BRCA* mutations [296]
			Phase II	NCT00494442	Olaparib demonstrated notable antitumor activity in advanced OC patients with *BRCA* mutations [297]
		BRCA-wild type recurrent OC	Phase II	NCT04091204	No results posted, recruiting patients
			Phase II	NCT05233982	No results posted, recruiting patients
		Platinum-sensitive relapsed OC with mutations in HR genes	Phase II	NCT02983799	Olaparib treatment was well-tolerated and demonstrated activity in *BRCA*-mutated non-*BRCA*-mutated, and HR-mutated OC patients [298]

		Platinum-sensitive relapsed OC	Phase II	NCT00753545	Platinum-sensitive relapsed or recurrent OC patients with *BRCA* mutations treated with olaparib had longer overall survival and progression-free survival [299,300]
		Advanced tumors with mutations in HR genes	Phase II	NCT03742895	No results posted
			Phase II	NCT03967938	No results posted, recruiting patients
		BRCA-mutated OC	Phase III	NCT01844986	The use of olaparib as a maintenance therapy improved progression-free survival and decreased risk of progression or death in advanced OC with *BRCA* mutations [301,304]
			Phase III	NCT02282020	Olaparib statistically improved objective response ratio and progression-free survival compared to non-platinum chemotherapy in platinum-sensitive relapsed OC patients with germline *BRCA* mutations that had received at least 2 prior lines of platinum-based chemotherapy [305]
			Phase III	NCT01874353	Olaparib as a maintenance treatment significantly improved progression-free survival in platinum-sensitive OC patients with germline *BRCA* mutations [306]
		BRCA-wild type OC	Phase III	NCT03402841	Maintenance olaparib treatment demonstrated clinical benefit in patients with non-mutated *BRCA* genes [307]
		BRCA-wild type advanced OC	Phase III	NCT04884360	No results posted, recruiting patients
		Relapsed OC	Phase III	NCT03534453	The maintenance treatment with olaparib demonstrated effectivity and being well-tolerated in platinum-sensitive relapsed Asian OC patients regardless *BRCA* status [308]
				NCT03106987	Olaparib maintenance treatment was safe and improved progression-free survival regardless *BRCA* status and a proportion presented a clinically relevant long-term benefit [309]
		OC	Phase III	NCT04421963	No results posted
		Recurrent OC	Phase III	NCT05255471	No results posted, recruiting patients

		Platinum-sensitive relapsed OC with *BRCA*-mutation	Phase IV	NCT04330040	Olaparib was well-tolerated and safety for Indian patients with platinum-sensitive relapsed OC in partial or complete response to platinum-base chemotherapy [310]
		BRCA- or HR-mutated genes OC	Phase IV	NCT02476968	Progression-free survival in patients with platinum-sensitive relapsed OC who received olaparib as maintenance treatment was similar regardless *BRCA* or *HR*-genes status [311]
		Cancer	Phase IV	NCT05078671	No results posted, recruiting patients
		Solid tumors	Observational	NCT02489058	No results posted
		OC	Observational	NCT04699006	No results posted
				NCT04553926	No results posted, recruiting patients
		BRCA-mutated platinum- sensitive relapsed OC	Observational	NCT02503436	Olaparib was well-tolerated under routine condition in this cohort of patients [312]
		BRCA-wild type OC	Observational	NCT05153603	Not yet recruiting
		BRCA-mutated platinum- sensitive recurrent OC	Observational	NCT02262273	No results posted
		Relapsed OC	Observational	NCT04152941	Efficacy and toxicity were like those obtained in NCT01874353 and NCT00753545 [313]
				NCT03505307	No results posted
		BRCA-mutated advanced OC	Observational	NCT04532645	Safety was like that obtained in previous reports of olaparib maintenance treatment [314]
		OC	Observational	NCT04560452	No results posted, recruiting patients
	Cisplatin + Paclitaxel + Bevacizumab	OC	Phase I	NCT02121990	Olaparib added to cisplatin/paclitaxel/bevacizumab was feasible [315]
	Carboplatin + paclitaxel	Relapsed OC	Phase I/II	NCT01650376	Olaparib/carboplatin/paclitaxel could be co-administered safely in pre-treated relapsed OC with 24% completed remission. *BRCA*-mutated patients had statistically significant longer progression-free survival and overall survival [316]

		Unresectable OC	Phase II	NCT04261465	No results posted, recruiting patients
		Platinum-sensitive advanced OC	Phase II	NCT01081951	Olaparib/paclitaxel/carboplatin combination followed by maintenance monotherapy significantly improved progression-free survival compared to paclitaxel/carboplatin, with a greater benefit in *BRCA*-mutated patients, and was well-tolerated [317]
	PLD	*BRCA*-mutated relapsed OC	Phase II	NCT00628251	Olaparib treatment was efficacy in *BRCA*-mutated advanced OC patients after a platinum-free survival lower than 12 months and was not statistically different from PLD-efficacy [318]
		Platinum-resistant persistant OC	Phase II	NCT03161132	No results posted
	Bevacizumab	Advanced OC	Phase III	NCT02477644	The addition of olaparib to bevacizumab maintenance therapy increased progression-free survival in patients with advanced OC specially in patients with mutations in *BRCA* and HR genes [319-322]
		BRCA-wild type OC	Observational	NCT05440578	Not yet recruiting
	Pembrolizumab	Advanced tumors with mutations in HR genes	Phase II	NCT04123366	No results posted, recruiting patients
		OC HR-mutated genes	Phase II	NCT04417192	No results posted, recruiting patients
		BRCA-wild type advanced OC	Phase III	NCT03740165	No results posted
	Pembrolizumab + bevacizumab	Platinum-sensitive recurrent OC	Phase II	NCT04361370	Olaparib/pembrolizumab/bevacizumab triple combination presented a potential benefit in *BRCA*-wild type patients. This combination showed a manageable safety profile [323]
			Phase II	NCT05158062	No results posted, recruiting patients
	Carboplatin	Recurrent or refractory women cancers	Phase I	NCT01237067	Pharmacokinetics of olaparib in presence or absence of carboplatin was assessed, recommending no-dose adjustment [324]

		OC	Phase I	NCT01445418	Olaparib/carboplatin combination was safe and presented activity in *BRCA*-mutated patients [325]
		Advanced cancers	Phase I	NCT02418624	Maximum tolerable dose of olaparib combined with two carboplatin cycles was established. This combination was feasible and relatively well-tolerated and showed a promising antitumor activity with a great decrease in tumor volume [326]
	Cobicistat	Cancer	Phase IV	NCT05078671	No results posted, recruiting patients
	Lurbinectedin	Advanced solid tumors	Phase I/II	NCT02684318	Lurbinectedin/olaparib combination was feasible and recommended doses were obtained for each drug [192]
	CYH33	Advanced solid tumors	Phase I	NCT04586335	No results posted, recruiting patients
	Abemaciclib	Platinum-resistant recurrent OC	Phase I	NCT04633239	No results posted, recruiting patients
	Alpelisib	*BRCA*-wild type platinum-resistant or refractory HGSC OC	Phase III	NCT04729387	No results posted, recruiting patients
	Anlotinib	Platinum-sensitive recurrent OC	Phase II	NCT04566952	No results posted, recruiting patients
	Cediranib	Recurrent OC	Phase I/II	NCT01116648	The addition of cediranib to olaparib treatment extended progression-free survival and overall survival in relapsed platinum-sensitive OC mainly in patients without *BRCA* mutations [327,328]
		Recurrent OC	Phase II	NCT02345265	No results posted
				NCT03314740	Cediranib/olaparib combination was active but was not superior to chemotherapy in heavily pre-treated platinum-resistant OC patients [329]
		OC	Phase II	NCT02681237	Cedinarib/olaparib activity varied according to the mechanism of PARPi-resistance [330]
				NCT02340611	No results posted

		Recurrent platinum-resistant OC	Phase II	NCT02889900	Cediranib/olaparib combination showed a clinical activity in heavily pre-treated *BRCA*-wild type platinum-resistant OC patients [331]
		Platinum-resistant OC	Phase II	NCT03117933	Cediranib/olaparib showed a greater efficacy than monotherapy treatments [332]
			Phase II/III	NCT02502266	No results posted
		Platinum-sensitive recurrent OC	Phase III	NCT02446600	Cediranib/olaparib demonstrated similar activity to standard chemotherapy in platinum-sensitive relapsed OC patients [333]. Patients with mutations in *BRCA* genes cediranib/olaparib treatment presented significant clinical activity [334]
		Relapsed OC	Phase III	NCT03278717	No results posted, recruiting patients
	Ceralasertib	Recurrent OC	Phase II	NCT03462342	The combination of ceralasertib and olaparib was well-tolerated. No objective response was observed; however, a signal of activity was observed and depended on *BRCA1* status [198]
		Recurrent OC	Phase II	NCT03579316	No results posted about ceralasertib/olaparib combination
		Gynecological cancers	Phase II	NCT04065269	No results posted, recruiting patients
		Advanced solid tumors	Phase II	NCT02576444	Ceralasertib/olaparib combination has demostrated preliminary activity in *ATM*-mutated tumors and in *BRCA*-mutated PARPi-resistant HGSC OC patients [199]
	Vistusertib/ capivasertib	OC	Phase I/II	NCT02208375	No results posted
	Copanlisib	Platinum-resistant recurrent OC	Phase II	NCT05295589	No results posted, recruiting patients
	Entinostat	Recurrent, platinum-refractory, or platinum-resistant OC	Phase I	NCT03924245	No results posted
	EP0057	Advanced OC	Phase II	NCT04669002	No results posted

	Onalespib	Metastatic or recurrent OC	Phase I	NCT02898207	No results posted
	Selumetinib	*RAS*-mutated OC	Phase I	NCT05554328	Olaparib/selumetinib combination was well-tolerated and showed promising preliminary antitumor activity in patients with mutations in *RAS* [335]
		RAS-mutated recurrent or persistent OC	Phase II	NCT03162627	Not yet recruiting patients
	NUV-868	Advanced solid tumors	Phase I/II	NCT05252390	No results posted
	Navitoclax	HGSC OC	Phase I	NCT05358639	Not yet recruiting patients
	BKM120/ BYL719	HGSC OC	Phase I	NCT01623349	BKM120/olaparib combination was feasible; however low dose of BKM120 should be administrated. Clinical benefit was observed regardless *BRCA* status [336]
	Adavosertib	Recurrent OC<br>Refractory solid tumors<br>Advanced solid tumors	Phase II<br>Phase I<br>Phase II	NCT03579316<br>NCT02511795<br>NCT02576444	Adavosertib in monotherapy or combined with olaparib demonstrated efficacy in patients with resistance to PARPi. Adavosertib/olaparib combination presented manageable toxicities [215-217]
	Durvalumab	OC	Phase II	NCT04644289	No results posted, recruiting patients
	Durvalumab+/- bevacizumab	Advanced solid tumors	Phase I/II	NCT02734004	Olaparib/durvalumab/bevacizumab demonstrated promising efficacy in platinum-sensitive OC patients without *BRCA* mutations. Olaparib/durvalumab and olaparib/durvalumab/ bevacizumab combinations were safe [337]
		Advanced OC	Phase II	NCT04015739	No results posted
			Phase III	NCT03737643	No results posted
	Durvalumab +/- cedinarib	Advanced or recurrent OC	Phase I/II	NCT02484404	No results posted, recruiting patients

	Durvalumab + tremelimumab	*BRCA*-mutated recurrent, resistant, or refractory OC	Phase II	NCT02953457	Olaparib/durvalumab/tremelimumab was safe and seemed to increase progression-free survival
	Durvalumab + UV1	*BRCA*-wild-type recurrent OC	Phase II	NCT04742075	No results posted, recruiting patients
	Tremelimumab	Persistent OC	Phase I	NCT02485990	No results posted
		BRCA-mutated recurrent OC	Phase I/II	NCT02571725	Olaparib/tremelimumab was tolerable in heavily pre-treated *BRCA*-mutated recurrent OC and demonstrated evidence of therapeutic effect [338]
		Platinum-sensitive recurrent OC	Phase II	NCT04034927	No results posted
**Niraparib**	-	OC	Phase I	NCT03551171	Niraparib was well tolerated, and the toxicity was manageable in Chinese OC patients [339]
		Advanced solid tumors	Phase I	NCT03359850	Niraparib dose was adjusted in patients with advanced solid tumors, including OC that presented normal hepatic function or moderate hepatic impairment [340]
		Solid tumors	Phase I	NCT00749502	No results posted
		Platinum-sensitive recurrent OC	Phase II	NCT03891576	No results posted, recruiting patients
		OC	Phase II	NCT04641247	No results posted
		Advanced OC	Phase II	NCT04284852	No results posted, recruiting patients
		Advanced relapsed OC	Phase II	NCT02354586	Niraparib demonstrated clinically relevant activity in heavily pre-treated OC patients, especially those platinum-sensitive with homologous recombination repair deficiency [341]
		Advanced relapsed OC	Phase II	NCT04392102	No results posted
		Unresectable OC	Phase II	NCT04507841	Neoadjuvant niraparib treatment showed efficacy and tolerable toxicity in *BRCA*-mutated or homologous recombination repair deficiency OC patients with unresectable tumors [342]

		Relapsed OC	Phase II	NCT03759587	The profile of safety was acceptable and similar to other studies [343]
		Advanced relapsed OC	Phase II	NCT03759600	Niraparib showed to be efficacy and safety in heavily pre-treated advanced relapsed OC patients with homologous recombination deficiency [344]
		Homologous recombination deficiency OC	Phase III	NCT05460000	Not yet recruiting
		Advanced OC	Phase III	NCT03709316	Niraparib prolonged progression-free survival regardless of chemotherapy response and biomarker status [345]
		Advanced OC	Phase III	NCT02655016	Niraparib had a similar efficacy in advanced OC patients that had been undergone to primary debulking surgery than those who had been treated with neoadjuvant chemotherapy or interval debulking surgery. Patients treated with neoadjuvant chemotherapy or interval debulking surgery and who presented visible residual disease presented the highest reduction in the risk of progression with niraparib as a maintenance treatment [346]
		Platinum-sensitive relapsed OC	Phase III	NCT03705156	No results posted
		Platinum-sensitive OC	Phase III	NCT01847274	Niraparib maintenance treatment presented clinical benefits in platinum-sensitive OC regardless of response to last platinum-based chemotherapy, and less time without toxicity symptoms [347-348]
		Relapsed OC	Phase IV	NCT03752216	No results posted
		OC	Phase IV	NCT04861181	No results posted, recruiting patients
		BRCA-wild type advanced OC	Phase IV	NCT05187208	Not yet recruiting
		OC	Observational	NCT04295577	No results posted, recruiting patients
		OC	Observational	NCT05021562	No results posted, recruiting patients

		OC	Observational	NCT04589039	No results posted, recruiting patients
		OC	Observational	NCT05583799	Not yet recruiting
		OC	Observational	NCT04986371	Not yet recruiting
		Platinum-sensitive recurrent OC	Observational	NCT04546373	No results posted
		Platinum-sensitive responsive recurrent OC	Observational	NCT04617470	No results posted, recruiting patients
		BRCA-wild type recurrent OC	Observational	NCT04785716	Niraparib as maintenance treatment has shown effectivity and a safe toxicity profile in patients with *BRCA* non-mutated recurrent platinum sensitive OC patients. The Individualized dosing was essential for minimizing the adverse events [349]
	Anlotinib	Platinum-resistant recurrent OC	Phase II	NCT04376073	Niraparib/anlotinib combination showed promising antitumor activity in patients with platinum-resistant recurrent OC [350]
		Recurrent OC	Phase II	NCT05130515	Not yet recruiting
		OC	Phase II	NCT05311579	No results posted, recruiting patients
		Platinum-sensitive recurrent OC	Phase II	NCT05385068	Not yet recruiting
	AsiDNATM	Platinum-sensitive relapsed OC	Phase I/II	NCT04826198	No results posted, recruiting patients
	+/- atezolizumab	Recurrent OC	Phase III	NCT03598270	No results posted
	Bevacizumab	Platinum-sensitive OC	Phase I/II	NCT02354131	Niraparib/bevacizumab was well tolerated and showed a promising clinical statistical activity in platinum-sensitive recurrent OC patients [351-353]
		Platinum-sensitive recurrent OC	Phase II	NCT04734665	No results posted, recruiting patients
		ARID1A-mutated recurrent OC	Phase II	NCT05523440	Not yet recruiting

		Advanced OC	Phase II	NCT03326193	Niraparib/bevacizumab as a first-line maintenance treatment showed promising results. This combination was safe, similar to safety profiles of monotherapy treatments [354]
		Advanced OC	Phase II	NCT05183984	No results posted, recruiting patients
		Platinum-resistant or refractory recurrent OC	Phase II	NCT04556071	No results posted
		Advanced OC	Phase III	NCT05009082	No results posted, recruiting patients
	Brivanib	Recurrent OC	Phase I	NCT03895788	No results posted
	+/- carboplatin/ bevacizumab/ dostarlimab/ paclitaxel	OC	Phase I/II	NCT03574779	Niraparib/bevacizumab/dostarlimab triple combination was tolerable and demonstrated clinical activity in platinum-resistant OC, most of the patients were *BRCA*- wild type and presented a normal homologous recombination repair [355]
	Cobinetinib +/- atezolizumab	Advanced platinum-sensitive OC	Phase I	NCT03695380	No results posted, recruiting patients
	Copanlisib	Recurrent OC	Phase I	NCT03586661	No results posted, recruiting patients
	Dostarlimab	*BRCA*-mutated OC	Phase I	NCT04673448	No results posted, recruiting patients
		Platinum-resistant OC	Phase II	NCT03955471	Niraparib/dostarlimab combination was safe; however, this combination presented a low overall ratio of response [356]
		Relapsed OC	Phase II	NCT05126342	Not yet recruiting
		Recurrent OC	Phase II/III	NCT03651206	No results posted, recruiting patients
		Recurrent OC	Phase III	NCT04679064	No results posted, recruiting patients
	Dostarlimab + platinum-base chemotherapy	Advanced OC	Phase III	NCT03602859	No results posted
	Elimusertib	Advanced OC	Phase I	NCT04267939	No results posted
	Etoposide	Platinum-resistant or refractory recurrent OC	Phase II	NCT04217798	No results posted, recruiting patients

	Everolimus	Advanced OC	Phase I	NCT03154281	No results posted
	Ganetespib	Platinum-sensitive OC	Phase II	NCT03783949	No results posted
	Gartisertib	PARPi resistant and recurrent OC	Phase I	NCT04149145	Not yet recruiting patients
	Neratinib	Advanced OC	Phase I	NCT04502602	No results posted, recruiting patients
	Oregomovab	Platinum-sensitive recurrent OC	Phase II	NCT05335993	No results posted, recruiting patients
	Pembrolizumab	Recurrent OC	Phase I/II	NCT02657889	Niraparib/pembrolizumab combination was tolerable with promising antitumor activity even in patients without homologous recombination deficiency or *BRCA* mutations [357]
	PLD	Advanced OC	Phase I	NCT01227941	No results posted
	Surgery	Platinum-sensitive recurrent OC	Phase II	NCT03983226	No results posted, recruiting patients
	ZN-c3	Platinum-resistant OC	Phase I/II	NCT05198804	No results posted, recruiting patients
**Talazoparib**	-	Advanced solid tumors	Phase I	NCT01286987	Talazoparib treatment presented antitumor activity and was well tolerated in patients with advanced solid tumors including OC [358]
		Advanced OC	Phase I	NCT02316834	No results posted
		BRCA-mutated OC	Phase I	NCT04598321	No results posted, recruiting patients
		Advanced solid or *BRCA*-mutated tumors	Phase I/II	NCT01989546	Talazoparib presented clinical activity and manageable toxicity in patients with advanced solid tumors or *BRCA*-mutated tumors, including OC
		BRCA-mutated OC	Phase II	NCT02326844	Only 3 patients were enrolled, talazoparib showed safe toxicity profiles
		BRCA-mutated recurrent, refractory,	Phase II	NCT02286687	Talazoparib demostrated clinical benefit in patients with alterations in genes of the *BRCA* pathway [359]

		advanced or metastatic tumors			
	Avelumab	*BRCA*- or *ATM*-mutated solid tumors	Phase II	NCT03565991	Talazoparib/avelumab combination was well tolerated and was efficacy mainly in *BRCA*-associated tumors, including OC. Its efficacy was similar to talazoparib efficacy in monotherapy [360]
		Advanced or metastatic solid tumors	Phase II	NCT03330405	Talazoparib/avelumab combination had an activity comparable with those with monotherapy treatment. A prolonged duration of the response was observed in *BRCA*-altered OC patients [361]
		Advanced OC	Phase III	NCT03642132	No results posted for talazoparib/avelumab combination
	Belinostat	Metastatic OC	Phase I	NCT04703920	No results posted, recruiting patients
	Radiation	Recurrent gynecological cancers	Phase I	NCT03968406	No results posted, recruiting patients
	ZEN003694	Recurrent OC	Phase II	NCT05071937	No results posted, recruiting patients
		Advanced solid tumors	Phase II	NCT05327010	No results posted, recruiting patients
**Pamiparib**	-	OC	Phase II	NCT05489926	No results posted, recruiting patients
		Advanced solid tumors	Phase I/II	NCT03333915	Pamparib showed antitumor activity with durable response in platinum-sensitive or platinum-recurrent OC patients with *BRCA* mutations and had a manageaple safety profile [362]
	Surufatinib	Platinum-resistant OC	Phase I/II	NCT05494580	Not yet recruiting
	Temozolomide	Advanced or metastatic tumors	Phase I/II	NCT03150810	Pamiparib/temozolomide combination was well tolerated and showed preliminary antitumor activity [363]
**Rucaparib**	-	Advanced solid tumors or *BRCA*-mutated OC	Phase I/II	NCT01482715	Rucaparib pharmacokinetics was characterized in patients with solid tumors including HGSC OC. Rucaparib was tolerable and showed activity in platinum-sensitive *BRCA*-mutated HGSC OC [364-365]

		Platinum-sensitive relapsed OC	Phase II	NCT01891344	Platinum-sensitive OC patients with *BRCA*-wild type, *BRCA* mutations, or homologous recombination deficiency treated with rucaparib presented a longer progression-free survival [366-367]
		BRCA-mutated advanced OC	Phase II	NCT00664781	No results posted
		Solid tumors with mutations in HR genes	Phase II	NCT04171700	No results posted
		OC	Phase III	NCT04227522	No results posted, recruiting patients
		OC	Phase III	NCT04676334	No results posted
		OC	Phase III	NCT01968213	Recurrent platinum-sensitive OC patients with two or more prior platinum-base chemotherapy, who were treated with rucaparib as maintenance treatment, presented a longer progression-free survival. Rucaparib treatment was safe and provided a clinical benefit even in patients without mutations of *BRCA* genes or low loss of genomic heterozygosity [368-370]
		BRCA-mutated OC	Phase III	NCT02855944	Rucaparib demonstrated to be an alternative treatment option to chemotherapy for patients with *BRCA*-mutated relapsed OC [371]
		OC	Observational	NCT04539327	Rucaparib treatment was safe and presented manageable toxicities [372]
	Atezolizumab	Advanced OC	Phase I	NCT03101280	No results posted
		Platinum-sensitive solid tumors or DNA reparir-deficient tumors	Phase II	NCT04276376	No results posted, recruiting patients
	Carboplatin + paclitaxel +/- bevacizumab	Advanced OC	Phase I/II	NCT03462212	The maximum tolerated dose of rucaparib was established when it was co-administrated with bevacizumab [373]
	Ipatasertib	Advanced OC	Phase I	NCT03840200	No results posted (for OC patients)

	Lucitanib or Sacituzumab govitecan (SG)	Advanced solid tumors	Phase I	NCT03992131	This study suggested an intermittent dosing of PARP inhibitors together with SG to reduce myelosuppression and optimize antitumor efficacy; however, further research is needed. Lucitanib/rucaparib combination had an acceptable safety profile [374-375]
	Mirvetuximab	Recurrent OC	Phase I	NCT03552471	No results posted
	Nivolumab	OC	Phase III	NCT03522246	Rucaparib treatment was effective as first-line maintenance, conferring a significant benefit in patients with advanced OC with or without homologous recombination deficiency [376]
	Nivolumab + bevacizumab	Relapsed OC	Phase II	NCT02873962	No results posted, recruiting patients
